# Phosphoregulation of Twist1 Provides a Mechanism of Cell Fate Control

**DOI:** 10.2174/092986708785908987

**Published:** 2008-10

**Authors:** Anthony B Firulli, Simon J Conway

**Affiliations:** Riley Heart Research Center, Herman B Wells Center for Pediatric Research, Division of Pediatric Cardiology, Departments of Anatomy and Medical and Molecular Genetics, Indiana Medical School, 1044 W. Walnut St., Indianapolis, IN 46202-5225, USA

**Keywords:** Twist1, bHLH, transcription, dimerization, DNA binding, Saethre Chotzen Syndrome, limb development, phosphorylation.

## Abstract

Basic Helix-loop-Helix (bHLH) factors play a significant role in both development and disease. bHLH factors function as protein dimers where two bHLH factors compose an active transcriptional complex. In various species, the bHLH factor Twist has been shown to play critical roles in diverse developmental systems such as mesoderm formation, neurogenesis, myogenesis, and neural crest cell migration and differentiation. Pathologically, Twist1 is a master regulator of epithelial-to-mesenchymal transition (EMT) and is causative of the autosomal-dominant human disease Saethre Chotzen Syndrome (SCS). Given the wide spectrum of *Twist1* expression in the developing embryo and the diverse roles it plays within these forming tissues, the question of how *Twist1* fills some of these specific roles has been largely unanswered. Recent work has shown that Twist’s biological function can be regulated by its partner choice within a given cell. Our work has identified a phosphoregulatory circuit where phosphorylation of key residues within the bHLH domain alters partner affinities for Twist1; and more recently, we show that the DNA binding affinity of the complexes that do form is affected in a cis-element dependent manner. Such perturbations are complex as they not only affect direct transcriptional programs of Twist1, but they indirectly affect the transcriptional outcomes of any bHLH factor that can dimerize with Twist1. Thus, the resulting lineage-restricted cell fate defects are a combination of loss-of-function and gain-of-function events. Relating the observed phenotypes of defective Twist function with this complex regulatory mechanism will add insight into our understanding of the critical functions of this complex transcription factor.

## THE BASIC HELIX LOOP HELIX PROTEIN

The bHLH domain is an evolutionarily conserved motif that is well represented from humans to flatworms. The bHLH domain consists of a short stretch of basic amino acids followed by an amphipathic α-helix, a loop of varying length and then another amphipathic α-helix (for detailed review see [1]). Each of the α-helices allows for protein-protein interactions with other bHLH proteins. The result of dimerization is the juxtaposition of the basic domains creating a combined DNA binding motif that in the majority of proteins allows for binding to a canonical sequence termed an E-box (CANNTG) [1]. Although HLH proteins can be classified into 5-subclasses, it is convenient to generalize categorization into 3 major classes: ubiquitously expressed bHLH factors (E-proteins Class A); tissue specific/restricted bHLH factors (Class B); and the negative regulatory HLH Id factors, which lack a basic DNA binding domain thereby sequestering E-proteins from forming functional transcriptional complexes [1]. Through the study of the Class B myogenic bHLH factors, it was established that these proteins could drive skeletal muscle specification and differentiation *via* heterodimer formation with bHLH factors from Class A [2-4]. Moreover, Id class HLH factors could compete for E-proteins as dimer partners adding a critical regulatory input to the system. As additional class B proteins were discovered, this regulatory model was initially applied; however, it became clear that not all Class B bHLH factors fit this simple paradigm.

## TWIST A bHLH FACTOR REQUIRED FOR MESODERM FORMATION 

In the fly, Twist was identified as a critical factor for the onset of gastrulation and the formation of mesoderm [5-7]. Regulated in part by Dorsal, Twist and the Zn-finger factor Snail coordinate with Dorsal to specify mesoderm in the fly. Mechanistically, it was presumed that Twist required a dimer partner from Class A to regulate gene expression [7]; however, in contrast to the established mechanism for the myogenic bHLH factors, Twist appeared capable of functioning as a homodimer. In elegant work from the Baylies laboratory, they showed that Twist conveyed different biological functions depending on the dimer partner choice. Using a tethered dimer approach to link Twist to itself or to Daughterless (the Class A E-protein in fly) *via* a short glycine linker sequence, the function of specific Twist dimer complexes were assayed. Expression of Twist-Twist homodimers in the fly resulted in mesoderm specification such that ectopic expression led to the formation of somatic muscle in inappropriate locations [7]. Moreover homodimer expression can rescue the early gastrulation defects in *Twist* mutant files. In contrast, Twist-Daughterless heterodimers antagonize mesoderm gene expression and genetic interactions show a complex gene dosage relationship [7]. These studies were the first to demonstrate that Class B bHLH factors could partner with a non-E-protein partner and facilitated a better understanding of the role played by one the vertebrate orthologs of Twist: Twist1. These studies also beg the question, how is dimer choice controlled?

## TWIST1 REGULATES MESENCHYMAL CELLS POPULATIONS IN MICE

**Fig. (1):**
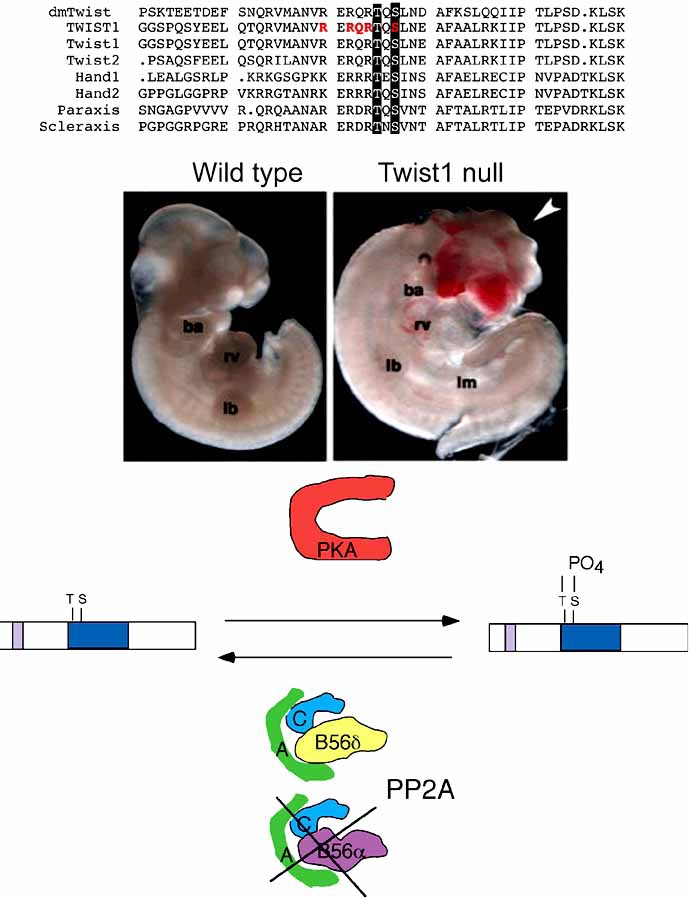
Regulatory conservation of Twist-family bHLH factors . Top shows amino acid alignment of human TWIST1 with murine protein family members Twist2, Hand1 and 2, Paraxis and Scleraxis. The conservation of the phosphoregulated threonine (T) and serine (S) is noted by black shading. Conservation is maintained back to invertebrates [25]. Red-bolded residues shown in the human sequence identify specific point mutations found within SCS patients. Middle panels show a wildtype and Twist1 null embryo at time of death E11.5. Note the pronounced exencephaly (white arrowhead), hypoplastic limb buds (lb), and reduced lateral mesoderm (lm). Bottom shows the phosphoregulatory circuit that governs Twist-family dimer control and DNA binding. PKA is capable of phosphorylation Twist1 whereas only PP2A complexes containing B56δ can specifically dephosphorylate the helix I resides.

Evolutionary conservation of critical proteins is well established between species. Given the importance of Twist in the fly, it seems logical that *Twist* orthologs would play equally important roles in higher organisms. Indeed, the identification of Twist-related factors shows the representation in higher species as well as in early organisms such as *C. elegans* and in mammals there are six Twist orthologs (Twist1, Twist2, Hand1, Hand2, Paraxis, and Scleraxis) [8-14] (Fig. **1**). 

In mouse, Twist1 function was directly assessed by gene deletion [15]. *Twist1* null embryos die around E11.5 and display a number of defects that reflect a functional role in mesenchymal cell populations. Major phenotypes include exencephaly, hypoplastic limb buds, and vascular defects [15] (Fig. **1**). These defects correlate to tissues that require cranial neural crest cells (NCC) to emigrate and contribute to the effected tissue [15, 16]. Our own data further shows that Twist1 also plays a role in mediating outflow track (OFT) cushion formation within the developing heart and that the defects observed in *Twist1* null OFTs result from defects in cardiac NCC cell behavior [17]. Recently, a conditional null *Twist1* allele has been reported and the use of this mouse model in looking at tissue-specific Cre deletions will shed additional light on all of the lineages that contribute to these phenotypes [18].

*Twist1* heterozygote null mice display a number of phenotypes including dysmorphic facial features and preaxial polydactyly in a partially penetrant fashion. Presentation of these phenotypes is dependent on mouse background and fits the gene dosage model established in the study of drosophila Twist. Interestingly, these haploinsufficient phenotypes are similar to an autosomal dominant, haploinsufficient disease in humans called Saethre Chotzen Syndrome (SCS). Not coincidently, a high percentage of SCS patients have null, mis-sense or non-sense mutations in *TWIST1* (see section below).

## TWIST1 AND SAETHRE CHOTZEN SYNDROME

SCS (OMIM101400) affects between 1-25,000 to 1-65,000 live births (for detailed review [19]). Amongst the phenotypic traits of SCS patients are craniosynostosis, low frontal hairline, facial asymmetry, and eyelid ptosis. Limb defects are also observed and include polydactyly, brachydactyly and syndactyly [20]. Although SCS can result from gene mutations in other factors, such as Snail [21, 22], the majority of documented SCS cases show a loss-of-function mutation in the human *TWIST* gene. Identification of *TWIST* was facilitated by the observations made in regards to the phenotypic similarities between SCS and *Twist1* heterozygous null mice as well as the fact that data shows SCS maps to 7p21-p22, which is homologous to mouse chromosome 12 region BC1, the location of *Twist1* [19]. To date, 73 known mutations in *TWIST* have been identified in SCS patients and although a number of these mutations involve large deletions, a number of mutations are point mutations that cluster near the basic DNA-binding domain. Initial presumption was that these mutants would affect DNA binding; however, DNA binding of this subset of *TWIST1* SCS alleles was subsequently established [11].

**Fig. (2):**
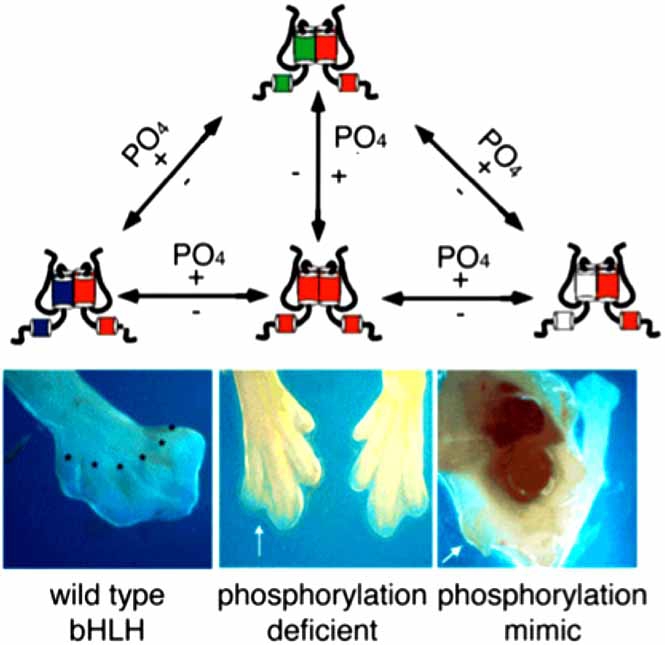
Model of Twist–family bHLH protein dimer regulation. Twist-family proteins have been shown to exhibit promiscuous dimerization characteristics that allow for multiple functional partners. In addition to expression levels of bHLH proteins within a cell as well as E-protein titration *via* Id factors, the phosphorylation state modulates Twist-family protein dimer affinities for its available partners thereby driving biological function. Expression of hypophosphorylation or phosphorylation mimic forms of the protein conveys distinct phenotypes in *vivo.* (Fig. (**2**) adapted from [24] PKA, PKC, and the Protein Phosphatase 2A Influence HAND Factor Function: A Mechanism for Tissue-Specific Transcriptional Regulation © 2003 with permission from Elsevier).

In the study of the Twist1-related proteins Hand1 and Hand2, it was also observed that these factors could form and function as non-E-protein dimers [23, 24]. Given that it was well established that Hand1 and Hand2 could and did function as heterodimers with E-proteins, the idea that homodimers could also convey biological function requires that dimer choice must be a regulated process. In an effort to determine how Hand dimer regulation was controlled, we uncovered a phosphoregulatory circuit involving protein kinase A (PKA) or PKC and the trimetric protein phosphatase 2A (PP2A) containing the B56δ regulatory subunit which could phosphorylate-dephosphorylate both Hand1 and Hand2 on a serine and threonine just carboxy to the basic domain [25] (Fig. **1**). Studies using phospho-deficient and phosphorylation mimic forms of Hand1 showed that changing the charge of helix 1 was sufficient to alter Hand1 affinities for its possible bHLH dimer partners. Moreover, when these Hand1 point mutants were ectopically expressed *in* *vivo*, distinct limb phenotypes were obtained [25] (Fig. **2**). Upon closer examination of the evolutionary conservation of these residues within the Twist-family, it was quickly determined that these residues were conserved in all Twist family members as far back as Drosophila [26]. When *TWIST* SCS alleles displaying point mutations within the basic domain were compared to the wild type *TWIST* allele, it was found that these mutants did disrupt the consensus PKA site. Moreover, we noted that a *TWIST1* mutation at S123 (relative to the human sequence) was sufficient to cause SCS and this residue was identical to the phosphoregulated serine in both Hand1 and Hand2 [26] (Fig. **1**).

## ALTERED PHOSPHOREGULATION OF TWIST1 CAN CAUSE SCS

Work done by a number of groups showed that ectopic expression of *Hand2* within the developing limbs in both mice and chick results in preaxial polydactyly [27, 28]. *Hand2* is expressed within the developing limb buds and is associated with an auto-regulation loop with the morphogen Sonic hedgehog (shh). *Shh* expression within the limb in part defines the zone of polarizing activity (ZPA), which imparts positional identity to the forming hand. Hand2 over expression expands expression of *Shh* resulting in ectopic ZPA formation and thus extra digits [27, 28]. Interestingly, *Twist1* haploinsufficiency phenocopies the *Hand2* gain-of-function phenotype suggesting that gene dosage and possible functional interactions between Twist1 and Hand2 are critical for modulating digit positional identity.

Indeed validating this hypothesis, dimer interactions between Twist1 and Hand2 can occur *in vivo* and partial coexpression within the developing limb, confirms biological relevance to the observed Twist1-Hand2 dimer formation [26]. To directly investigate if phosphoregulation of Twist1 modulated Twist1 dimer choice, Fluorescence Resonance Energy Transfer (FRET) [29] was used to assay dimer interaction strength of Twist1 with itself, ubiquitous E12, and Hand2 [26]. Results of these studies show that wild type and phosphorylation mimic Twist1 displayed similar affinities for itself, E12 and Hand2 albeit at altered interaction strengths [26]. In contrast, the Twist1 hypophosphorylation mutant (which models an established SCS *TWIST1* allele) showed a distinct dimer affinity profile from the wild type protein, suggesting that TWIST1 dimer choice within a cell would be different dependent upon phosphorylation state [26]. Given that hypophosphorylated Twist1 displayed altered dimerization characteristics from wild type Twist1, phosphorylation analysis of the basic domain *TWIST1* SCS alleles was undertaken. As predicted, these mutations showed a decreased ability to be phosphorylated by PKA *in vivo* supporting the idea that phosphoregulation of these evolutionarily conserved threonine and serine residues can modulate the biological activity of Twist1 [26]. Considering that 5 independent *Twist1* SCS point mutations encode proteins with a reduced ability to be phosphorylated and that hypophosphorylated Twist1 displays distinct preferences for various bHLH partners, the idea that this molecular switch modulates Twist1 function is appealing.

## TWIST1 AND HAND2 DISPLAY ANTAGONISTIC FUNCTION IN THE LIMB

In examining the Twist1 FRET interaction data, the interactions with Hand2 are most divergent. For instance, wild-type Twist1 has the highest interaction affinity for Hand2, whereas the SCS helix 1 hypophosphorylation Twist1 mutant has the lowest affinity for Hand2 dimerization [26]. This observation, in addition to the observation that Twist1 loss-of-function phenocopies Hand2 gain-of-function in regards to polydactyly, led us to conduct a genetic test of this intriguing biochemical model. The experiment was a simple intercross of a* Hand2* null allele onto a *Twist1* haploinsufficient background, thus taking what was effectively a *Hand2* gain-of-function (2 *Hand2* alleles to 1 *Twist1* allele) and rebalancing the gene dosage to one copy of each bHLH partner. The results of this experiment show a complete rescue of polydactyly on the *Twist1* heterozygous background [26]. In similar studies in the chick using retrovirus over expression, *Hand2* expression results in polydactyly, which can be partially rescued *via* coexpression of retrovirus expressing wild-type *Twist1*. In contrast, coexpression of a SCS helix 1 hypophosphorylation *Twist1* mutant retrovirus fails to rescue *Hand2* generated polydactyly [26]. These findings support the hypothesis that Twist1 dimer choice is regulated by the actions of PKA and B56δ-containing PP2A and can convey a distinct biological function to Twist1. As these residues are also conserved in Drosophila, Twist phosphoregulation likely controls dimer choice in this genetic model system.

Interpretations are complicated when partner choice has many inputs: how do you interpret results and what is the best experiment?

What is still not clear from this data is the identity of the specific dimer pairs that are regulating specific molecular programs. Within a given cell, multiple bHLH and HLH factors are coexpressed temporally and in a dynamic fashion. The obvious changes in stoichiometry by altering ratios of any bHLH protein will affect the availability of E12 and other factors that can find each other and dimerize. The expression of Id factors further complicates this relationship as Id factors can titrate available E-proteins levels directly. By this logic, over expression of bHLH factors must be viewed in a different light. Swamping a cell with many more copies of one factor will undoubtedly result in E-protein titration, unintended bHLH heterodimers, and over expressed homodimers that will collectively orchestrate many of the resultant phenotypes. Even in “simple” gene knockout studies, the removal of a bHLH transcription factor will clearly result in the loss of regulation of downstream target genes; additionally, the dimer pools within the cell will be altered allowing for the formation of a new dimer pool that will contain bHLH complexes that would not normally form and thus modulate gene expression in unintended ways. Simply put, any gene knockout of a factor that requires a partner for biological activity is very likely to exhibit phenotypes that include direct loss-of-function and deleterious gain-of-function mechanisms. This is exemplified by the observation that *Twist1-Hand2* double heterozygous null mice are more phenotypically normal than mice heterozygote for only *Twist1*.

As Fig. (**3**) schematizes, the balance between *Twist1* and *Hand2* within the developing limb is critical for normal morphogenesis and that phosphoregulation of Twist1 influences this relationship, thus an increase in Hand2 relative to Twist1 results in polydactyly. Would the gene dosage manipulation work in the opposite direction? That is, would having more *Twist1* relative to *Hand2* also produce abnormal development? In gain-of-function experiments, wild type, hypophosphorylation and phosphorylation mimic forms of Twist1 were expressed within the developing limbs of mice using the limb specific *Prx1* promoter [30]. Results show that Twist1 gain-of-function resulted in medial defects within both the fore- and hindlimbs; however, as predicted by the gene dosage model, no polydactyly was observed. What was observed is that the phosphorylation mutants display unique phenotypes. Consistent with Twist1T125; S127A being an SCS allele, it shows a less severe phenotype then wildtype Twist1 [30]. Given that hypophosphorylated Twist1 shows a reduced antagonism for Hand2, this data fits the model well. Interestingly, the Twist1 phosphorylation mimic shows the most dramatic limb phenotypes including a severe reduction in ossification and medial limb structures; but again, no polydactyly was observed. Clearly, Twist1 gain-of function is mediating limb defects that are distinct from those of Hand2 gain-of-function.

One must consider that these are gross over expression experiments and given that the presumed mechanism is dimer formation, we cannot account for the deleterious titration of endogenous bHLH factors that would result in their altered function. One obvious solution to decoding the mechanism underlying these observed phenotypes is to employ the experimental approach used in the study of drosophila twist and employ tethered dimers to look at direct downstream effects. Mouse Twist1 tethered proteins bind DNA and transactivate promoters in a manner similar to when Twist and E12 are expressed as separate polypeptides [30, 31]. When expressed in the developing limb, distinct phenotypes for Twist1-Twist1 homodimers, Twist1-E12, and Twist1-Hand2 heterodimers are observed and those phenotypes correlate well with the phenotypes observed by the expression of the monomeric wild type and mutant Twist1 proteins [30]. Interestingly, the expression of Twist1 homodimers displayed similar limb phenotypes to those observed by the expression of the Twist1 phosphorylation mimic. Twist1-E12 tethered dimers show similar defects as those exhibited by the expression of wildtype Twist1. Most surprisingly, Twist1-Hand2 tethered complexes showed polydactyly and a mild loss of some medial structure; a combinatorial effect supporting the possibility of more then antagonistic functions in the limb program. Although the phenotypes are clearly not identical, the differences observed between the monomeric and tethered dimer data likely reflect the effect of endogenous bHLH factor titration from monomer over expression that will not occur when using a tethered dimer pair.

To complicate the mechanism still further, it has also been shown in the monomeric analysis that phosphoregulation of Twist1 influences its affinity for E-boxes in a *cis-*element dependent manner [30]. Thus in addition to dimer choice, phosphorylation influences which E-box elements that the Twist1-containing bHLH complexes will bind. In combination with chromatin remodeling, which is the ultimate dictator of transcription factor accessibility, a highly regulated scheme emerges where the overall level of bHLH expression within a cell, combined with the phosphoregulation of the Twist bHLH family members will define a Twist-family dimer pool within that cell. This dimer pool will then drive transcriptional programs based on the ability of the Twist dimers formed to access compatible *cis*-elements available for interaction. Id factors, which will independently influence the amount of E-protein available, also convey dimer choice by a simple swing of mass action. Thus, amphipathic protein structures need to interact to be stable in an aqueous environment and a dramatic change in the access of one will greatly influence the interactions of the others.

It is interesting to consider how sensitive biological programs are to this elaborate regulatory mechanism. How many molecules of one factor vs. another will tip the balance between modulating normal vs. abnormal gene expression? How much do post-translational modifications modulate this critical dosage? Although we cannot yet answer these questions, we can see examples within the same animal where such issues must be at play. For example, the Hand2 transgenic shown in Fig. (**3**) displays asymmetrical polydactyly despite the observation that the *Prx1*-promoter does not show asymmetrical expression levels between left and right [32]. Does this result reflect a threshold of Hand2 expression that was reached in one but not the opposing limb and/or a variation in phosphorylation state of either Twist1 or Hand2 at a critical point in development? Addressing these questions would require more elegant *in vivo* and *in vitro* experimental systems and analysis. To avoid issues of over expression, direct helix I point mutant knockins for both *Twist1* and *Hand2* would allow for a better assessment of gene dosage within the tissues that need to specifically express these factors. Although tethered dimer knockin animal models would be more artificial, the use of a conditional activation allele expressing such a tethered complex could add valuable insight within specific developmental windows that would lead to a better understanding of the role that Twist1 plays within the mesenchymal cell populations that allow for the complex body structure in multi-cellular organisms.

## TWIST AND CANCER

In addition to its essential role in modulating the behavior of mesenchymal cell populations critical for development, *Twist1* is also an oncogene and is associated with a number of aggressive neoplasias including gastric, liver and most notably breast cancers [33-38]. The oncogenic role of Twist1 is not in facilitating cell transformation but rather it facilitates the ability of the cells within a primary tumor to undergo a pathological EMT similar to its function in development. EMT allows tumor cells to migrate away from the primary tumor, enter the lymphatic system, and settle into secondary tumor sites or metastasis [37]. Using a mouse mammary tumor model, Yang and colleagues made use of 4 tumor cell lines isolated from the same mammary tumor that displayed distinct abilities to promote metastasis in mice. Subtractive screens identified Twist1 expression as being a predictor of metastaic behavior and the study goes on to show that the most aggressive metastaic cell line could be rendered non-metastaic by siRNA knockdown of Twist1 expression [37]. Conversely, using a gain-of-function approach they show that expression of Twist1 in epithelial cell lines drives EMT making the cells mesenchymal in phenotype [37]. Taken together, this data suggests Twist1 as a master regulator of EMT. In the developing embryo it allows for cell migration programs critical for normal body patterning; whereas in cancer, it allows for secondary tumor formation, which is the ultimate cause of mortality.

## FUTURE DIRECTIONS

The pivotal role that Twist1 plays in both embryonic development and disease is well established. In both of these roles, the biological function of Twist1 within mesenchymal cell populations is obvious. In comparison to the remaining Twist-family members, other then loss-of-function phenotypes resulting from targeted gene deletion, Twist1 is the only protein within the family that displays dominant disease phenotypes. It is likely that other family members play critical roles in utero and the lack of evidence for these factors contributing to postnatal disease may reflect phenotypes that result in early embryonic death. Suspiciously, all family members are expressed within tissues that undergo morphology changes. Has gene expansion through evolution allowed for more specialized functions regulating cell shape and behavior? Currently this is our favorite hypothesis, which we are in the process of testing. Point mutant knockins for the various Twist-family members are underway and should shed insight into such cell behavior.

Of note, when considering the role of Twist1 in cancer progression, is the observation that although *Twist1* appears necessary for metastasis in the mouse breast cancer model, it is probably not sufficient given that 3 of the 4 cell lines express comparable levels of Twist1 protein yet 2 of the 3 cell lines are largely non-metastatic [36]. Given that Twist1 protein levels are similar yet metastatic behavior is different, an additional component to Twist1 functional regulation must be required for metastasis. It will be interesting to investigate the role of Twist1 phosphoregulation in the process of tumor progression thus linking the elaborate control of dimer choice and DNA binding preferences to neoplastic disease. In support of this hypothesis, PP2A, has recently been identified as a tumor suppressor [39] and B56δ containing PP2A complexes could play a role in regulating Twist1 function in cancer *via* control of the phosphorylation state of Twist1. If Twist1 regulation *via* phosphorylation is indeed a critical component of tumor progression, it will provide a potential therapeutic target to inhibit EMT thereby reducing the incidence of lethal pathologies. Further investigations into gaining a better understanding of the Twist-family functional mechanism will likely add valuable insights into the roles that this transcription factor family plays in development and disease.

## Figures and Tables

**Fig. (3):**
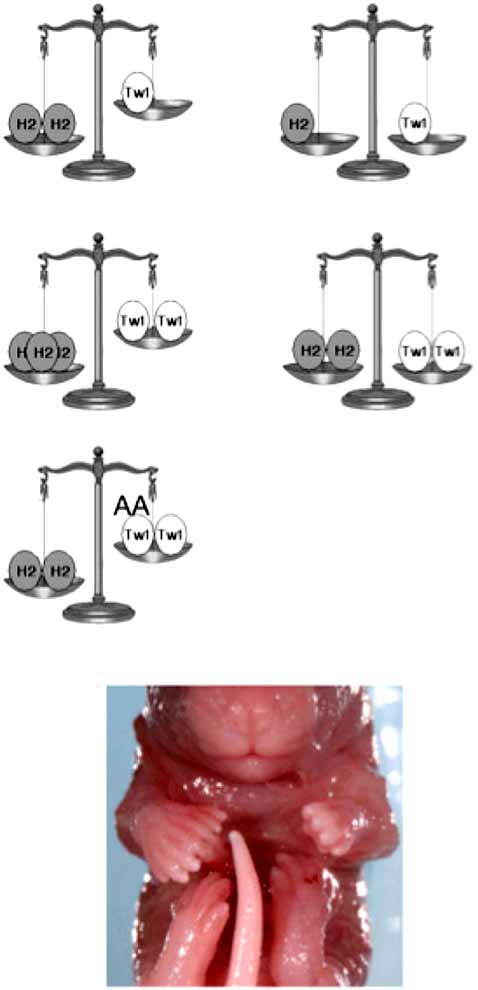
Gene balance model between *Twist1* and *Hand2* in the developing limb. Left shows genotypes that convey Twist1 haploinsufficiency resulting in polydactyly where as genotypes to the right convey normal limb development. Of note, point mutations that disrupt phosphorylation (Twist1T125;S127A: TW1AA) of Twist1 result in phenotypes indistinguishable from a genetic imbalance with *Hand2.* Below is an E17.5 day transgenic mouse embryo expressing Hand2 *via* the Prx1-limb-specific promoter. Obvious is right forepaw polydactyly with left forepaw showing normal digit formation. Given that Prx1-expression *via* this promoter fragment is not asymmetric [31], this example shows the critical balance of Twist-Hand2 gene dosage as subtle differences in expression between left and right limbs within the same animal can result in different phenotypes.
